# IS26-Flanked Composite Transposon Tn6539 Carrying the *tet*(M) Gene in IncHI2-Type Conjugative Plasmids From *Escherichia coli* Isolated From Ducks in China

**DOI:** 10.3389/fmicb.2018.03168

**Published:** 2019-01-15

**Authors:** Ya-wei Sun, Ying-ying Liu, Hua Wu, Ling-fei Wang, Jian-hua Liu, Li Yuan, Yu-shan Pan, Dan-dan He, Gong-zheng Hu

**Affiliations:** ^1^Department of Pharmacology and Toxicology, College of Animal Husbandry and Veterinary Science, Henan Agricultural University, Zhengzhou, China; ^2^Department of Animal Science, Henan Institute of Science and Technology, Xinxiang, China

**Keywords:** composite transposonTn6539, *tet*(M) gene, IS26 element, IncHI2-type plasmid, *Escherichia coli* isolates

## Abstract

Tet(M)-type proteins confer resistance to tetracycline and related antibiotics by interacting with the ribosome. Genes encoding Tet(M) have been found in a range of bacteria, including *Escherichia coli*. In the current study, conjugation experiments were performed between seven different tetracycline-resistant, azide-susceptible *E. coli* strains isolated from ducks and tetracycline-sensitive, azide-resistant *E.coli* J53. Transconjugants were obtained from two of the strains at a frequency of 1.2 × 10^−8^. PCR, southern blotting and sequencing demonstrated that *tet*(M) in the transconjugants was located on a ~50 kb IncHI2-type plasmid and was part of a composite transposon, designated Tn6539. This transposon is flanked by two IS26 elements in opposite orientation and contains the Tn3Δ*tnpA*+Δ*orf13*-*lp*-*tet*(M)+gamma delta+*tnpX*+Δ*tnpR* sequences. The Δ*orf13*-*lp*-*tet*(M) sequence was a highly conserved genetic fragment in *E. coli* harboring *tet*(M) and mainly located in the composite transposons flanked by IS6-family elements. In summary, Tn6539 is a new composite transposon capable of horizontal transfer of *tet*(M) among *E. coli* isolates.

## Introduction

Resistance to tetracycline and related antibiotics can be conferred by efflux pumps, enzymatic inactivation and ribosomal protection proteins such as the ones encoded by *tet*(M). Tet(M) confers a wider spectrum of resistance to tetracyclines than efflux proteins except for Tet(B) and has the widest host range of any tetracycline resistance gene (Chopra and Roberts, 2001; Roberts and Schwarz, 2016). Currently, *tet*(M) has been found in more than 60 genera (http://faculty.washington.edu/marilynr/), likely because of the association of *tet*(M) with integrative and conjugative transposons, which facilitate horizontal transfer. For example, tet(M) was found to be associated with Tn916/Tn1545-like conjugative transposons in *Enterococcus* spp. (Cauwerts et al., 2007). Additionally, *tet*(M) was also found in Tn5801-like conjugative transposons in *Staphylococcus aureus* and Tn5397-like transposons in *Clostridium difficile* and *Enterococcus faecium* (Agersø et al., 2006; Vries et al., 2009). A *tet*(M) homolog was not found in clinical isolates of *Escherichia coli* until 2004 (Bryan et al., 2004), but has been described since then in isolates from different sources (Jones et al., 2006; Tuckman et al., 2007; Jurado-Rabadán et al., 2014; Gilrane et al., 2017). The *E. coli* isolates carrying *tet*(M) not only conferred resistance to oxytetracycline, tetracycline and doxycycline, but also to minocycline (Jones et al., 2006; Hu et al., 2013). At the same time, Tet(M) protein has the potential to acquire mutation leading to increased MICs of tigecycline (Linkevicius et al., 2015). Therefore, the spread of *tet*(M) has made tetracycline treatment for *E. coli* infections a clinical dilemma. However, which mobile genetic elements facilitate horizontal transfer of *tet*(M) among *E. coli* isolates is still poorly understood. To the best of our knowledge, only one report has described a mobile element containing *tet*(M) flanked by IS26 and ISVs1 in human (Jones et al., 2006).

Our previous study revealed that *tet*(M) plays a role in doxycycline resistance of *E. coli* isolated from ducks in China (Hu et al., 2013). In the current study, seven of the previously isolated doxycycline-resistant strains were used in conjugation experiments and in two cases, transfer of *tet*(M) was apparently mediated by a plasmid that harbored a new composite transposon, designated Tn6539. The structure of this transposon was elucidated by PCR, cloning, and sequencing experiments.

## Materials and Methods

### Bacterial Strains

Seven full-length *tet*(M)-positive *E. coli* isolates CY4, E5, W4, LF6, Y8, CY14, and 5Y (Table 1) containing part of *orf13*, the promoter regions of *tet*(M), the Tet(M) leader peptide gene, and intact *tet*(M) were previously isolated from ducks (Hu et al., 2013). *E. coli* J53 and *E. coli* DH5a were used as the recipient strain in mating experiment and the host strain in cloning experiments, respectively.

**Table 1:** Bacterial strains used in this study.

**Strain**	**Source/location**	**Multilocus sequence type**	**Phylogenetic grouping based on multiplex PCR**	**Genetic sequence /move genetic element carrying *tet*(M)**	**GenBank accession number**
CY4	Dead duck/Henan province	ST48	A	ΔIS26-Δ*orf13*-*lp*-*tet*(M)	KJ772289
5Y	Dead duck/Henan province	ST156	B1	Tn6539-like	MF422120
E5	Dead duck/Guangdong province	ST3839	D	ΔIS26-Δ*orf13*-*lp*-*tet*(M)	KJ772289
W4	Dead duck/Fujian province	ST162	B1	Tn6539	KJ772290
LF6	Dead duck/Fujian province	ST224	A	Δ*orf13*-*lp*-*tet*(M)	JF830611
Y8	Dead duck/Zhejiang province	ST163	B1	Δ*orf13*-*lp*-*tet*(M)	JF830611
CY14	Dead duck/Zhejiang province	ST224	B1	Δ*orf13*-*lp*-*tet*(M)	JF830611

### Conjugation Experiments

Seven *E. coli* isolates harboring full-length *tet*(M) were used for filter mating experiments with sodium azide-resistant *E. coli* J53 as a recipient as previously described (Devirgiliis et al., 2009). Briefly, overnight cultures of tetracycline-resistant, sodium azide-susceptible donor (*E. coli* isolate) and tetracycline-susceptible, sodium azide-resistant recipient (*E. coli* J53), grown in Luria-Bertani (LB) broth, were seeded at a 1:50 dilution in separate flasks of fresh LB broth. Following growth to log phase (OD_600_≈0.6) with shaking at the speed of 250 rpm at 37°C, 0.5 ml each of the donor and the recipient cultures were mixed and filtered through a 0.22-μm-pore-size membrane (Millipore Corp.) placed on prewarmed on MacConkey agar plates. After 24 h of incubation at 37°C, the cells were detached from filters in 5 ml of 1 × phosphate-buffered saline (pH7.4), and serial dilutions were plated on MacConkey agar plates containing 16 mg/L doxycycline and 100 mg/L sodium azide. Controls (unmixed donors and recipient cells) were treated in the same manner. Transconjugant colonies were recovered following incubation at 37°C for 24 to 72 h. Furthermore, amplification of the sequences of *tet*(A), *tet*(B), *tet*(C), and *tet*(M) in the transconjugants were performed using the primer sets in Table 2. Template DNA was prepared as follows. Amounts of 1.5 ml of transconjugant culture were pelleted, and cells were boiled in 200 μl of H_2_O for 15 min. After centrifugation, the supernatants were kept at −20°C. PCR was performed in a total volume of 50 μl, which contained 3 μl of supernatant, 50 pmol of each primer, 25 μl of PrimeSTAR® Max DNA Polymerase (TakaRa Biotechnology Co. Ltd, Japan). After an initial denaturation step of 3 min at 95°C, amplification was performed over 30 cycles, each one consisting of 30 s at 95°C, 30 s min at hybridization temperature [55°C for *tet*(A) and *tet*(C), 58°C for *tet*(B), and 52°C for *tet*(M)], and 1 min at 72°C, with a final extension step of 10 min at 72°C. The amplicons were sequenced using their corresponding primer set at commercial company (Shanghai Sangon Biological Engineering Technology and Services Co. Ltd, China). The sequences comparisons were performed using the BLASTtool available online at the National Center for Biotechnology Information of the National Library of Medicine (http://www.ncbi.nlm.nih.gov/blast/). Transconjugants carrying *tet*(M), but not *tet*(A), *tet*(B), or *tet*(C) were selected for further analysis.

**Table 2:** Primers used in this study.

**Primer**	**Sequence (5′-3′)**	**Start point(bp)^a^**	**Amplicon size (bp)**	**Use and notes**	**Reference sequence accession no^b^**
*tet*(A) F	GCTACATCCTGCTTGCCTTG	797–816	210	Sequence of *tet*(A)	NC 015599
*tet*(A) R	CATAGATCGCCGTGAAGAGG	987–1006			
*tet*(B) F	TTGGTTAGGGGCAAGTTTTG	396–415	695	Sequence of *tet*(B)	MF969100
*tet*(B) R	GTAATGGGCCAATAACACCG	1035–1054			
*tet*(C) F	CTTGAGAGCCTTCAACCCAG	579–598	418	Sequence of *tet*(C)	NG 048181
*tet*(C) R	ATGGTCGTCATCTACCTGCC	977–996			
F1	GGTCATCAACACGGATAAAGC	2163–2183	1761	Sequence between *IS26* and *tet*(M)	KJ772290
F2	ATGTCCTGGCGTGTCTATGAT	3903–3923			
F4	AGAAATCCCTGCTCGGTGTAT	5426–5446	987	Sequence between *tet*(M) and *tnpX*	KJ772290
F5	GATGTTACTGGCTTGGTTTGA	6392–6412			
F6	CTTCATTTCCTATCGGTATCT	6242–6262	1887	Sequence between *tnpX* and *tnpR*	X60200
F7	AAGGTGTATCCATTCGGTTTA	8108–8128			
F8	CAACAACTCCTTTTGCCATT	7967–7986	1004	Sequence between Δ*tnpR* and reversed *IS26*	LO017738
F9	GAAGCAAATAGTCGGTGGTG	8951–8970			
F10	GCCCTATCCTACAGCGACAG	3073–3092	2500	Sequence between Δ*orf13* and *tet*(M)	KJ772290
F11	ATCCGACTATTTGGACGACG	5553–5572			
tetM-F	GTGGACAAAGGTACAACGAG	3795–3814	406	Sequence in *tet*(M)	KJ772290
tetM-R	CGGTAAAGTTCGTCACACAC	4181–4200			

a *Numbering is the sequence of KJ772290*.

b *GenBank accession numbers (www.ncbi.nlm.nih.gov)*.

### Antimicrobial Susceptibility Testing

The susceptibility of *tet*(M)-positive transconjugants, their corresponding donors, and *E. coli* J53 to different antibiotics (Table 3) was determined by broth microdilution assays (Clinical Laboratory Standards Institute, 2014). Minimum inhibitory concentrations were determined on three independent occasions. *E. coli* ATCC 25922 was used for quality control in all susceptibility tests.

**Table 3:** Characteristics of strains used in conjugation experiments.

**Strains**	**MICs (unit: mg/L)**									***tet* genes detected**	**Plasmid replicon types detected**
	**OXY**	**TET**	**DOX**	**AMK**	**NEO**	**CF**	**CTX**	**FLO**	**ENR**		
J53	1	1	0.5	1	4	0.25	0.125	2	<0.25	ND	ND
W4	>512	512	128	>512	512	64	8	256	128	*tet*(A), *tet*(B), *tet* (C*) tet*(M*)*	HI2, FIB, Y, P, and A/C
TW4	256	128	16	>512	512	0.5	<0.5	64	1	*tet*(M)	HI2
5Y	>512	512	64	512	512	128	<0.5	256	128	*tet*(A*), tet*(B), *tet*(C*) tet*(M*)*	HI2, P, and A/C
T5Y	128	128	32	512	512	0.5	<0.5	64	0.5	*tet*(M)	HI2

*OXY, oxytetracycline; TET, tetracycline; DOX, doxycycline; AMK, amikacin; NEO, neomycin; CF ceftiofur; CTX, cefotaxime; FLO, florfenicol; ENR, enrofloxacin; TW4 and T5Y is the tet(M)-positive transconjugant of E. coli isolate W4 and 5Y, respectively. ND, not tested*.

### Plasmid Analysis

The incompatibility (Inc) groups of plasmids from transconjugants and their corresponding donors were determined by PCR-based replicon typing (Carattoli et al., 2005). Plasmids from the transconjugants and their corresponding donors (W4 and 5Y) were extracted using the QIAGEN Plasmid Midi Kit (Hilden, Germany) and subjected to electrophoresis on a 0.7% agarose gel. Plasmid size was estimated by comparison with reference plasmid DNAs of *E. coli* V517. The purified plasmids from W4 and 5Y were transferred to a nylon membrane (Roche Molecular Systems, Basel, Switzerland) and Southern blotting was carried out using a digoxigenin-labeled *tet*(M) PCR fragment [406 base pairs (bp), Table 2] as a probe.

### Sequence Determinations

The *tet*(M)-positive conjugative plasmid pTW4 was digested with *Eco*RI and the fragments were ligated into the *Eco*RI-digested pBluescriptIISK(+). The recombined plasmids were electroporated into *E. coli* DH5α and white colonies were selected on LB/X-gal/IPTG agar supplemented with ampicillin (100 mg/L) and oxytetracycline (128 mg/L). The presence of *tet*(M) in transformants was confirmed by PCR using the primer set tetM-F and tetM-R (Table 2). The plasmid inserts were sequenced using M13 universal sequencing primers. The nucleotide and deduced protein sequences were analyzed with EditSeq and Megalign software (DNAstar, Madison, WI, USA). Sequence similarity and conserved domain searches were carried out using the BLASTtool available online at the National Center for Biotechnology Information of the National Library of Medicine (http://www.ncbi.nlm.nih.gov/blast/).

### PCR Mapping Analysis

Based on the sequence of the obtained inserted fragment between *hp* and partial *tnpX* (6,544 bp, the left sequence of KJ772290 in **Figure 2**) and the complete sequence of Tn1000 (accession number X60200), the primer set F6 and F7 (Table 2) was designed to amplify the sequence between *tnpX* and the truncated *tnpR*. The amplicon was ligated to the 3′-terminus of the inserted fragment. The sequence between partial *tnpR* sequence (Δ*tnpR*) and right-terminal IS26 element was amplified using the primer set F8 and F9 designed according to the corresponding sequence of plasmid PRC557 (accession no. LO017738).

To monitor whether similar genetic organization exists in the six remaining *E. coli* isolates, PCR mapping was performed using the primer set F6 and F7, F8 and F9, and the primer sets designed using the software Primer Primer 5.0 (PREMIER Biosoft, American) on the obtained sequence in the plasmid pTW4 (accession no. KJ772290). The primer sets are shown in Table 2, **Figure 2**. All PCR amplicons were sequenced using their corresponding primer set at commercial company (Shanghai Sangon Biological Engineering Technology & Services Co. Ltd, China), and the results were compared to sequences in the GenBank database using the BLAST tool.

### Molecular Typing and Phylogenetic Analysis

The seven *E. coli* isolates were typed by multilocus sequence typing (MLST) (http://mlst.warwick.ac.uk/mlst/) as previously described (http://www.shigatox.net/mlst) (Clermont et al., 2015). Briefly, seven housekeeping genes (*aspC, clpX, fadD, icdA, lysP, mdh*, and *uidA*) were amplified and sequenced. The corresponding sequence types (STs) were matched using the electronic database on the *E. coli* MLST website. Additionally, the phylogenetic groups of the seven isolates were determined based on PCR detection of the *chuA* and *yiaA* genes and DNA fragment TSPE4.C2 (Clermont et al., 2000).

### Nucleotide Sequencing and Submission of *tet*(M) Sequences

The nucleotide sequences containing full-length *tet*(M) were deposited in GenBank (accession numbers KJ772290, KJ772289, and MF422120).

## Results

### Transfer of *tet*(M) and Multidrug Resistance

Two *tet*(M)-positive transconjugants (TW4 and T5Y) were obtained from donors W4 and 5Y at a frequency of 1.2 × 10^−8^. As shown in Table 3, W4, 5Y, TW4, and T5Y exhibited resistance to tetracycline, oxytetracycline, and doxycycline. The minimum inhibitory concentrations of TW4 and T5Y to the above-mentioned drugs were 8- to 256-fold higher than those of the recipient strain *E. coli* J53. TW4 and T5Y also exhibited resistance to amikacin, neomycin, and florfenicol, suggesting that the corresponding resistance markers were co-transferred into the recipient strain. In addition, the transconjugants carrying *tet*(A) and *tet*(C) were also found on the selection plates.

### Molecular Characteristics of Conjugative Plasmids

**Figure 1:**
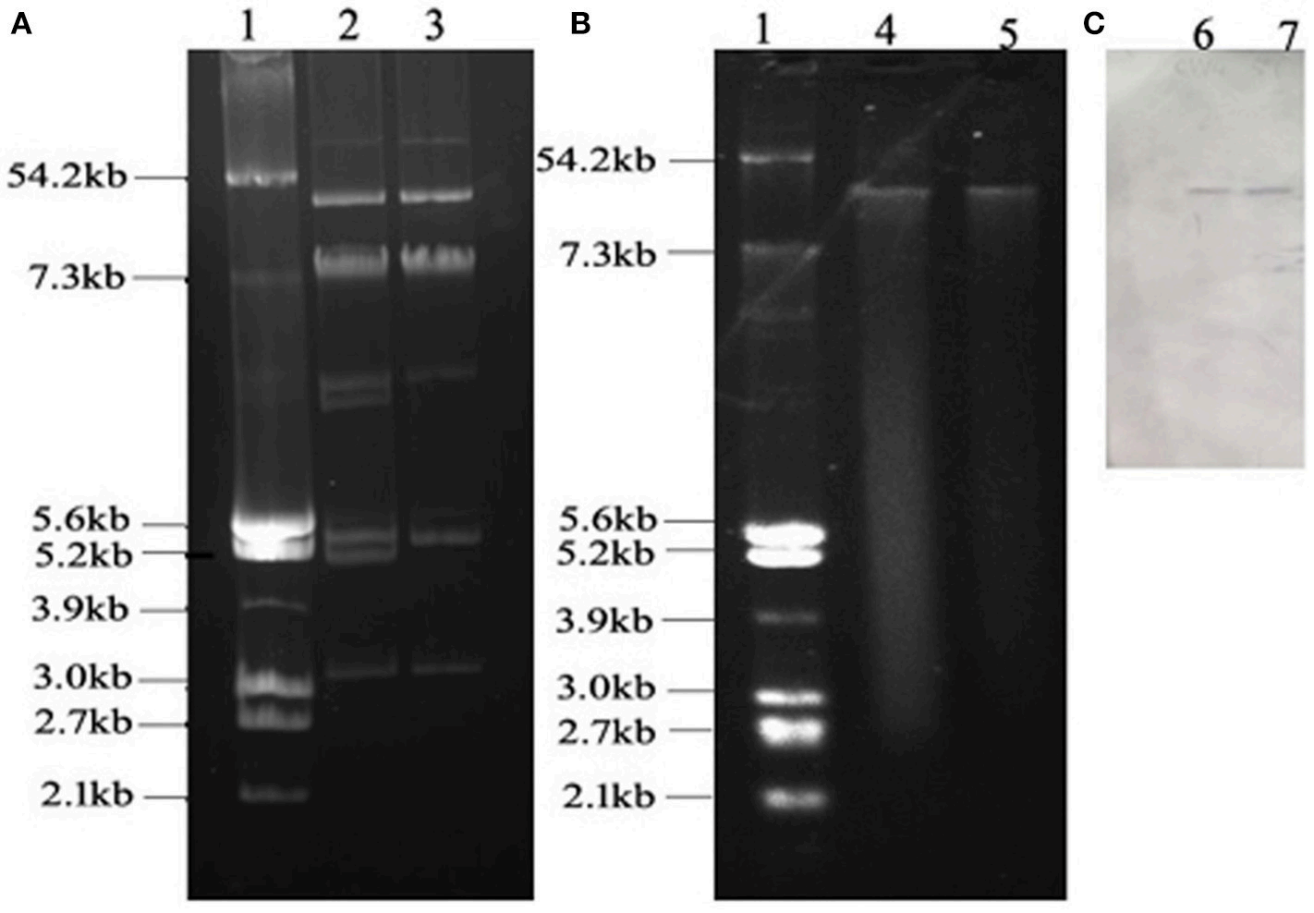
**(A)** Agarose electrophoresis of plasmids from *E. coli* strains W4 and 5Y. **(B)** Agarose electrophoresis of plasmids from transconjugant *E. coli* TW4 and T5Y. **(C)** Southern blot of plasmids from W4 and 5Y and labeled *tet*(M) fragment. Lane 1, reference plasmid DNAs of *E. coli* V517; lane 2, plasmids from W4; lane 3, plasmids from 5Y; lane 4, plasmid from TW4; lane 5, plasmid from T5Y; lane 6, southern blot, plasmids from W4; lane 7, southern blot, plasmids from 5Y.

W4 and 5Y carried more than three plasmids (Figure 1). The sizes of the plasmids ranged from approximately 3.2 to more than 54.2 kb. Notably, TW4 and T5Y each carried only one plasmid (designated pTW4 and p5Y, respectively) of approximately 50 kb. Southern hybridization with the digoxigenin-labeled *tet*(M) PCR fragment indicated that isolates W4 and 5Y yielded one distinct signal located on this plasmid. PCR-based inc replicon typing showed that pTW4 and p5Y belonged to the HI2 type. The other plasmids in W4 and 5Y were positive for the HI2, FIB, Y, P, and A/C and the P, and A/C type, respectively.

### Genetic Organization of *tet*(M) in Conjugative Plasmid and Sequence Analysis

**Figure 2:**
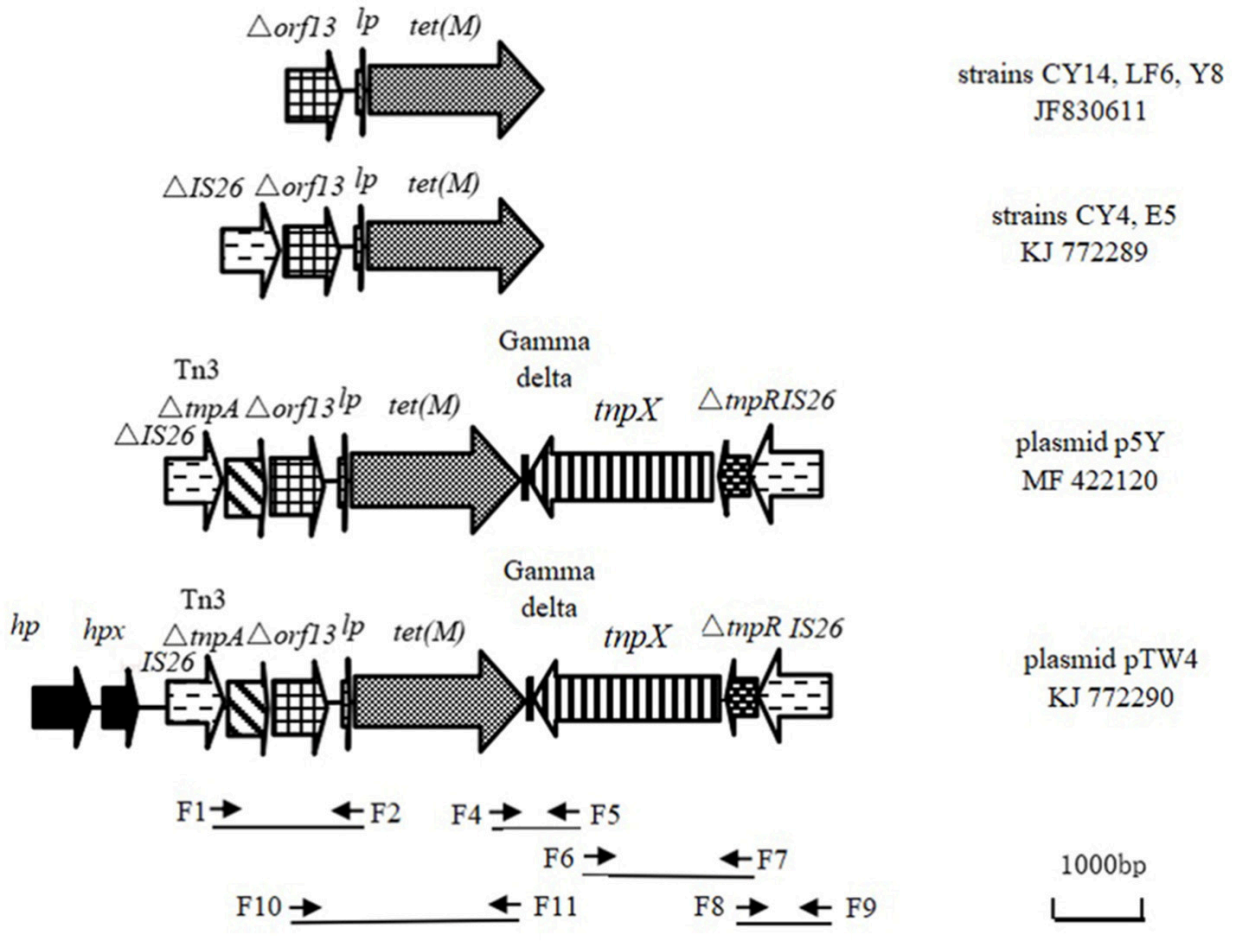
Location of *tet*(M) in clinical isolates of *E. coli* and graphical representation of primer pairs along the tested genetic structure KJ772290 (*hp*), gene encoding hypothetical protein; *hpx*, gene encoding putative protein X; *lp*, gene encoding Tet(M) leader peptide; fragment between A and B position in the sequence KJ772290 was obtained by cloning experiments. Arrows represent the orientation of each primer and relative positions of the primers along the tested linear sequence.

The analysis of recombinant plasmids carrying *Eco*RI fragments from the conjugative plasmid pTW4 demonstrated that *tet*(M) was located on a fragment of approximately 6.5 kbp between *hp* and truncated *tnpX* (pTW4 plasmid in Figure 2). Sequence analysis indicated that a truncated orf13, the *tet*(M) leader peptide gene and *tet*(M) are located between bp 2989 and 5609 (numbering nucleotide position starts from the first base, G, in the sequence of KJ772290.). Sequence comparisons showed that the Δ*orf13*-*lp*-*tet*(M) exhibited ≥99% sequence identity to the corresponding sequences present in the GenBank database. Among them, the Δ*orf13*-*lp*-*tet*(M) exhibited 100% sequence identity to the corresponding sequence in the chromosome of *Straphylococcus rostri* (accession no. FN550102), *Streptococcus* spp. (CP008813 and CP003859), *E. faecalis* DENG (CP004081), and *E. coli* (CP021844).

At the left end of the *tet*(M)-containing *Eco*RI fragment, codons 1–1,067 were indistinguishable from those found in plasmid pM160133 (CP022165) and plasmid pSCE516-1 (KX023262) from *E. coli* isolates from a New York patient and chickens in China. These isolates contained two ORFs in the same orientation as *tet*(M) encoding a hypothetical protein and putative protein X. Additionally, codons 2522–5609 showed 100% sequence identity to the corresponding sequences in the chromosome of *E. coli* (CP021840 and CP021844), which contain an ORF in the transcription orientation of *tet*(M) in addition to the Δ*orf13*-*lp*-*tet*(M). This ORF located in the left of Δ*orf13*-*lp*-*tet*(M) in the plasmid pTW4 was indistinguishable from the corresponding sequence of the 3′-terminal of the gene encoding Tn3-family transposase (TnpA) found in plasmid pM160133 (CP022165). Notably, an intact IS26 element including 14-bp perfect terminal inverted repeats and IS26 transposase was found between the gene encoding putative protein X and truncated Tn3 *tnpA*.

At the right end of the *tet*(M)-containing *Eco*RI fragment (basepairs 2522 to 65544), Δ*orf13*-*lp*-*tet*(M)+gamma delta +incomplete *tnpX* were present in pTW4 and these sequences were 99% identical to the corresponding sequence in plasmids p41-3 (LC318054) and p15 (LC317981) from *E. coli* isolates from beef cattle in Japan. Obviously, the downstream of Δ*orf13*-*lp*-*tet*(M) is a Tn1000 element. To determine whether a complete Tn1000 was present, we designed a forward primer for the obtained *tnpX* sequence and reverse primers for different positions along the sequence of Tn1000 (X60200). A fragment, 1,887 bp in size, between *tnpX* and Δ*tnpR* was obtained using primer set F6 and F7 (Table 2). Alignment of Δ*orf13*-*lp*-*tet*(M) to part of the sequence of Tn1000 suggests that an unknown mobile genetic element is present upstream of Δ*tnpR*. IS26 elements typically exist in genomic DNA by forming regions containing antibiotic resistance genes flanked by two IS26 elements in the same or opposite orientations (Naas et al., 2001; Harmer and Hall, 2016). Since an intact IS26 element was detected in the *tet*(M)-containing EcoRI fragment, we further extended the sequence at its 3′-terminal end and obtained a fragment of 1,004 bp using primer set F8 and F9 (Table 2). Therefore, pTW4 contains IS26+Tn3Δ*tnpA*+Δ*orf13*-*lp*-*tet*(M)+gamma delta+*tnpX*+Δ*tnpR*+ reverse IS26 (Figure 2). The presence of two intact IS26 elements suggests that the genetic organization carrying *tet*(M) is a previously unknown composite transposon which has now been registered as Tn6539 in the Transposon Nomenclature Database from UCL Eastman (http://transposon.lstmed.ac.uk/tn-registry).

### Genetic Sequences Carrying *tet*(M) in Six Remaining Strains of *E. coli* and Homology Analysis

The genetic sequences carrying *tet*(M) in the tested strains are shown in Figure 2. Four sequences from seven *E. coli* isolates carry the genetic fragment of Δ*orf13*-*lp*-*tet*(M). Among them, genomic DNAs from CY14, LF6, and Y8 only carry Δ*orf13*-*lp*-*tet*(M) which showed 99.1% nucleotide sequence identity to our previously obtained the sequence carrying *tet*(M) [JF83061]. A truncated IS26 element was located upstream of Δ*orf13* in the genomic DNA from CY4 and E5 (KJ772289). Additionally, the conjugative plasmid p5Y harbors a highly similar composite transposon to that in pTW4.

**Figure 3:**
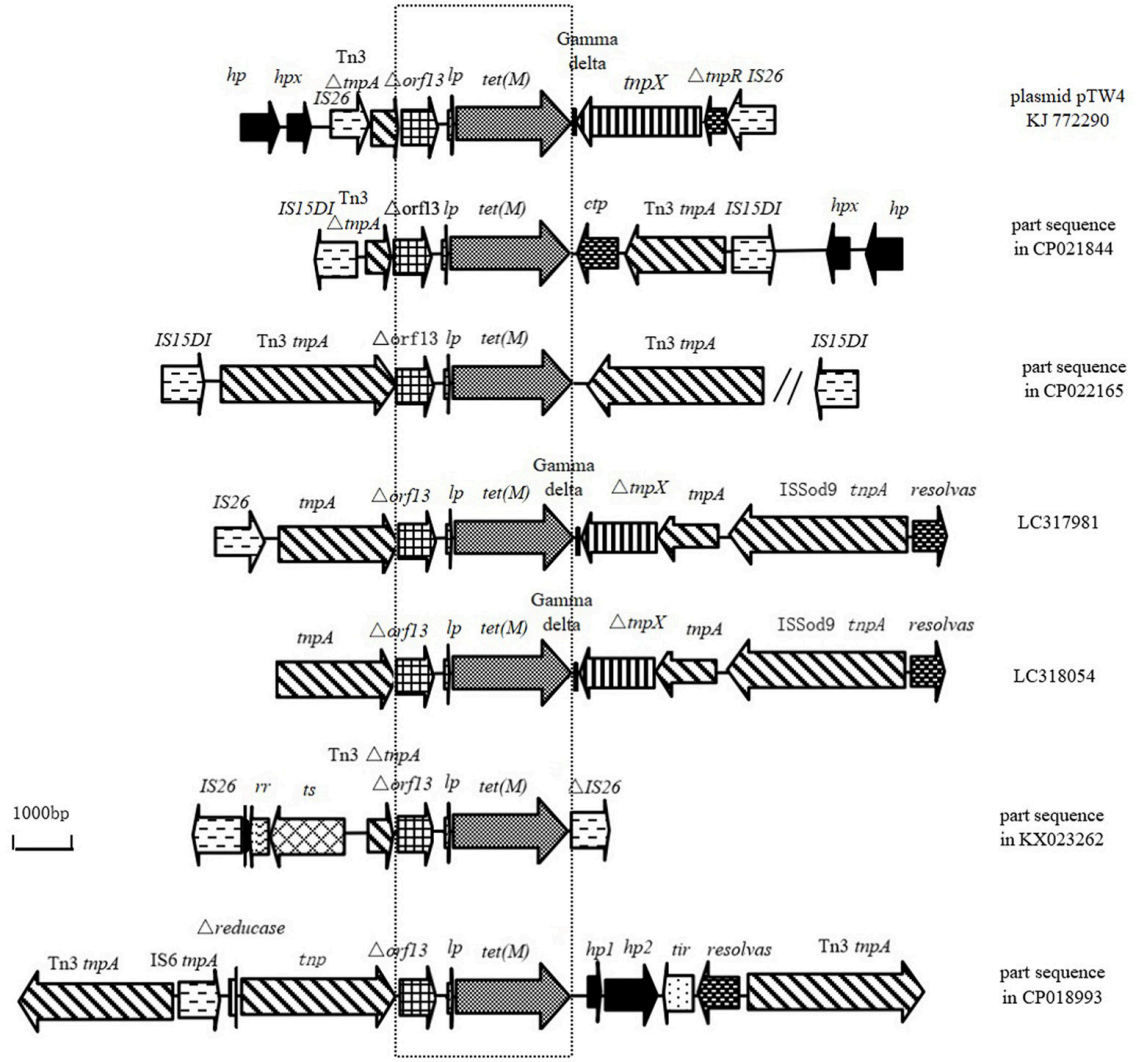
Structural features surrounding the sequence of Δ*orf13* +*lp*+*tet*(M) in KJ772290 compared to other sequences deposited in GenBank. (Similar regions are indicated by dotted lines; *hp*, gene encoding hypothetical protein; *hpx*, gene encoding putative protein X; *lp*, gene encoding TetM leader peptide; *ctp*, gene encoding conjugative transfer protein; symbol “//” the sequence of 13,102 bp between Tn3 tnpA and IS15D1; *rr*, gene encoding RadR regulator; ts, gene encoding chromate transporter).

The similarities of Tn6539 in plasmid pTW4 with the sequences from *E. coli* deposited in GenBank are shown in Figure 3. The Δ*orf13*-*lp*-*tet*(M) fragment is located upstream or at the 3′-terminal end of part or the complete gene encoding Tn3-family transposase in the genomic DNA from the six *E. coli* isolates. The genomic DNAs from five of the six isolates carry intact IS6–family element(s). Among them, the chromosomal DNA from *E. coli* EC974 (CP021844) harbors a composite transposon consisting of *orf13*-*lp*-*tet*(M) and two IS15DIV elements belonging to the IS6-family in opposite orientations at its boundaries. Additionally, an Inc HI2–type plasmid pM160133 (CP022165) also carries a composite transposon with a similar genetic origination to that in the chromosome of *E. coli* EC974. The length of the genetic fragment flanked by two IS15DIVs elements was 22,182 bp and carried the resistance genes *aadA2, dfrA12*, and *floR* in addition to *tet*(M).

### Genetic Relatedness and Phylogenetic Analysis of Seven *E. coli* Isolates

MLST analysis showed that the seven tested isolates had six different sequence types. W4, 5Y, CY4, E5, and Y8 belong to ST162, ST156, ST48, ST3839, and ST163, respectively. CY14 and LF6 belong to ST224. Notably, E5 belongs to a new MLST profile (ST3839). Phylogenetic analysis showed that the seven strains belonged to group A (CY4 and LF6), group B1 (W4, 5Y, Y8, and CY14), and group D (E5) which is commonly considered as including pathogenic bacteria (Clermont et al., 2000).

## Discussion

In this study, *tet*(M) was found in IncHI2-type conjugative plasmids from *E. coli* isolates W4 and 5Y. Although *tet*(M)-like genes are most commonly found in bacterial chromosome (Agersø et al., 2006; Vries et al., 2009), they also exist in conjugative plasmids from *Campylobacter jejuni* (Taylor et al., 1987), *Neisseria meningitidis, Kingella denitrificans, Eikenella corrodens* (Knapp et al., 1988), *Clostridium perfringens* (Yras and Rood, 1996), and *E. coli* (CP022165). Therefore, conjugative plasmids are an important vehicle facilitating the horizontal transfer of *tet*(M)-like genes between and across genera. IncHI2 plasmids play an important role in the acquisition and dissemination of antibiotic resistance genes among Gram-negative bacteria (Cain and Hall, 2012) and have been found to carry numerous classes of resistance genes including those conferring resistance to β-lactams (*bla*_SHV_, *bla*_CTX−M_, *bla*_CMY_, *bla*_OXA_, *bla*_TEM_, and *NDM-1*), quinolones [*oqxAB, qnrA, qnrS, qnrB*, and *aac(6*′*)-Ib-cr*], aminoglycosides (*armA, aadA, aacA4, strA*, and *strB*), amphenicols (*catA1* and *floR*), trimethoprin (*dhfr1*), sulfonamides (*sul1* and *sul2*), fosfomycin (*fosA3*), and colistin (*mcr-1*) (Chen et al., 2016; Fang et al., 2016; Hadjadj et al., 2017). Additionally, these multidrug-resistant IncHI2-type plasmids are broadly distributed among different bacteria including *E. coli, Salmonella* spp., *Shigella flexnei, Klebesiella* spp., and *Enterobacter cloacae* (Liu et al., 2015; Kieffer et al., 2017). Thus, the IncHI2-type conjugative plasmids carrying *tet*(M) found in this study may spread between and across genera in the future, threatening tetracycline treatment in clinical practice.

IS26, a member of the IS6 insertion sequence family, contributes to the dissemination of antibiotic resistance genes in Gram-negative bacteria mainly by forming composite transposons. Most composite transposons contain a center region harboring resistance genes and two IS26 elements in the same or opposite orientations at its bounders (Harmer and Hall, 2016). In Gram-negative bacteria, composition transposons are usually found with IS26 elements at their borders, but not flanked by 8-bp target site duplication (TSD), a hall marker of IS26 integration (Curiao et al., 2011; Zienkiewicz et al., 2013). Therefore, researchers considered that the composite transposons were formed by homologous recombination rather than a transposition. In 2014, a new model for IS26-mediated mobilization of resistance genes has been proposed by Harmer et al. (2014), whereby an IS26 element together with resistance gene(s), called a translocatable units (TU), is considered as a new family mobile genetic element. TU released from the genetic sequences can recognized another IS26 as target and is incorporated immediately adjacent to it in the same orientation. This results in IS26 element arraying in tandem and the formation of composite transposons. The frequency of cointegrate formation mediated by TU was 60-fold higher than that mediated by a single IS26, which indicated that an intact IS26 element in genetic sequences owns a stronger ability to recruit another IS26-mediated resistance genes. Furthermore, it was confirmed that TU integration could occur by Tnp (transposase in IS26 element)-catalyzed reaction or RecA-dependent homologous recombination. However, Tnp-catalyzed reaction was 100-fold more efficient than RecA-dependent homologous recombination (Harmer and Hall, 2015). In addition, He et al. (2015) analyzed the distinct patterns of sequences flanking 70 IS26 copies in eight genomes from Carbapenemase-producing *Enterobacteriaceae* and found that IS26 promoted rearrangement of resistance plasmids by both inter- and intramolecular replicative transposition (He et al., 2015). In the current study, Tn6539 was found to be a composite transposon containing Δ*orf13*-*lp*-*tet*(M) and two IS26 elements in opposite orientations at its borders. This type of composite transposon was first found in a multidrug-resistant plasmid from an *E. coli* MG-1 and named Tn2000. The center region flanked by two IS26 elements in Tn2000 is a class 1 integrin, In*53*, carrying nine functional resistance gene cassettes (Naas et al., 2001). Structures of composite transposons similar to that of Tn6539 were found in plasmids from *E. coli* isolates from different sources (Literacka et al., 2009; Smet et al., 2010; Yang et al., 2014), but the molecular mechanism of their movement among Gram-negative bacteria remains unknown and requires further analysis.

## Conclusion

We described the organization of a novel composite transposon Tn6539 in an IncHI2-type conjugative plasmid from two *E. coli* isolates with different genetic backgrounds. The transposon carries *tet*(M) and two intact IS26 elements in opposite orientations at its bounders.

## Author Contributions

GH and LY conceived and designed the experiments. YS, YL, HW, and LW performed the experiments. JL, YP, and YS analyzed the data. DH and HW contributed reagents materials analysis tools. YS and GH wrote the paper.

### Conflict of Interest Statement

The authors declare that the research was conducted in the absence of any commercial or financial relationships that could be construed as a potential conflict of interest.
